# Near infrared emission properties of Er doped cubic sesquioxides in the second/third biological windows

**DOI:** 10.1038/s41598-018-36639-y

**Published:** 2018-12-21

**Authors:** Daniel Avram, Ion Tiseanu, Bogdan S. Vasile, Mihaela Florea, Carmen Tiseanu

**Affiliations:** 1 National Institute for Laser, Plasma and Radiation Physics, P.O. Box MG-36, RO 76900 Bucharest, Magurele Romania; 2 University of Bucharest, Faculty of Physics, 405 Atomistilor Street, 077125 Magurele, Ilfov Romania; 3 University POLITEHNICA from Bucharest, National Research Center for Food Safety, 313 Splaiul Independentei Street, RO 060042 Bucharest, Romania; 4 National Institute of Materials Physics, 405A Atomistilor Street, 077125 Magurele, Ilfov Romania

## Abstract

In the recent years, there is an extensive effort concentrated towards the development of nanoparticles with near-infrared emission within the so called second or third biological windows induced by excitation outside 800–1000 nm range corresponding to the traditional Nd (800 nm) and Yb (980 nm) sensitizers. Here, we present a first report on the near-infrared (900–1700 nm) emission of significant member of cubic sesquioxides, Er-Lu_2_O_3_ nanoparticles, measured under both near-infrared up-conversion and low energy X-ray excitations. The nanoparticle compositions are optimized by varying Er concentration and Li addition. It is found that, under ca. 1500 nm up-conversion excitation, the emission is almost monochromatic (>93%) and centered at 980 nm while over 80% of the X-ray induced emission is concentrated around 1500 nm. The mechanisms responsible for the up-conversion emission of Er - Lu_2_O_3_ are identified by help of the up-conversion emission and excitation spectra as well as emission decays considering multiple excitation/emission transitions across visible to near-infrared ranges. Comparison between the emission properties of Er-Lu_2_O_3_ and Er-Y_2_O_3_ induced by optical and X-ray excitation is also presented. Our results suggest that the further optimized Er-doped cubic sesquioxides represent promising candidates for bioimaging and photovoltaic applications.

## Introduction

Among the rare earth oxides, cubic Lu_2_O_3_ is an attractive phosphor host for lanthanide (Ln) activators due to its high mass density (9.4 g cm^−3^), good phase stability, low thermal expansion, low phonon energy (phonon cutoff ~600 cm^−1^), and broad optical transparency from the visible to the near infrared (NIR) regions^1–4^. The top of the valence-band energy levels of Lu-based oxides are mainly composed of lutetium 4f orbital, whereas in Y-based materials, the corresponding top levels are mostly oxygen 2p orbital. Such a difference was considered to make Lu-based compounds more favorable than the Y-based ones as up conversion hosts^5^. The smaller cell volume may also enhance the energy transfer efficiency between lanthanide dopants in Lu_2_O_3_ in comparison with that in isostructural Y_2_O_3_ for the same fractional doping concentration^2^. There are already a few reports that suggest that the up-conversion emission intensity is up to two order of magnitude stronger in Lu_2_O_3_ compared to Y_2_O_3_ phosphor^3,6–8^. Besides, several investigations on Ln doped Lu-based compounds, such as Tm–Ho doped LuLiF_4_, and Tm/Er doped NaLuF_4_, have been proven to have stronger luminescence and better laser performance than their corresponding Y homologues^9–11^.

Despite that Lu_2_O_3_ is regarded, in principle, more advantageous than Y_2_O_3_ as an up-conversion host, only 17 articles were found by searching Scopus and ISI database in the last 10 years using “Lu_2_O_3_” and “up-conversion” as keywords compared to almost 100 for Y_2_O_3_. Among the Ln ions used in the up-conversion studies, Er single doped or co-doped with Yb sensitizer, represents the most investigated activator (12 articles) due to its multiple emission and absorption transitions spanning from ultraviolet (UV) to near infrared (NIR) spectral range.

Vetrone *et al*. showed^6^ that up-conversion under excitation (980 nm) of nanocrystalline 2%Er-Lu_2_O_3_ is lower than its bulk counterpart. Beside the presence of the ground state absorption (GSA) followed by excited state absorption (ESA) mechanisms, the contribution of the energy transfer up-conversion (ETU) mechanism was confirmed by the emission decays measurements both bulked and nanoparticulate samples. Capobianco *et al*.^3^ observed that under down-conversion excitation at 488 nm, the relative integrated intensity of the ^4^I_11/2_-^4^I_15/2_ (around 980 nm) and ^4^I_13/2_-^4^I_15/2_ (around 1500 nm) emission transitions is about 3:1 for bulked sample compared to 1:1 in nanocrystalline sample due to the presence of the carbonates and hydroxyl groups on the nanocrystalline surface. In addition, the same authors suggested that the much stronger (up to two orders of magnitude) up-conversion emission intensity measured with Er-Lu_2_O_3_ compared to Er-Y_2_O_3_ nanoparticles under 980 nm excitation was related to reduced adsorption of CO_2_ and H_2_O on the Lu_2_O_3_ surface induced by the greater particle size of Lu_2_O_3_ (50 nm) than Y_2_O_3_ (20 nm).

Recent research has shown that extending fluorescence imaging into shortwave IR (1000–2000 nm) wavelengths can further enhance the advantages of NIR imaging^12–16^. Low levels of background tissue autofluorescence in the shortwave IR increase imaging sensitivity to a target nanoprobe, and the unique tissue absorption and scattering properties increase contrast of structures at greater penetration depths compared with fluorescence imaging in the NIR^17–21^. On the other side, it is well known that the efficiency of solar cells is increased by converting the photons with energies below the absorption threshold (λ > 1100 nm) into higher energy photons (λ < 1100 nm)^22^. Such interesting applications explain the recent growing number of studies on Er based systems that present a relative intense up-conversion emission centered at 980 nm following excitation at 1500 nm, such as Er-CeO_2_^23,24^, Er-Y_2_O_3_^25^ and Er-NaYF_4_^26^, Er(Yb)-Gd_2_O_2_S^27–29^, Er-BaTiO_3_^30^, Er-BaY_2_F_8_^31^ and Er-LiYF_4_^32,33^. To the best of the authors knowledge, only one study investigated so far, the up-conversion emission of Er-Lu_2_O_3_ nanoparticles using ca. 1500 nm excitation^8^. However, the measured up-conversion emission included only the green emission (520–580 nm), disregarding the NIR emission around 980 nm. Following an extensive literature on Scopus database, we have found that, while in the last 10 years more than a 1000 studies have been published on the up-conversion emission of Er(Yb) doped systems under 980 nm excitation, less than 100 studies concerned 1500 nm as up-conversion excitation wavelength. Of these, less than 20 studies measured the 980 nm emission in Er(Yb) nanoparticulate systems. Finally, to the best of our knowledge, less than 10 studies report on (almost) pure/monochromatic up-conversion emission around 980 nm (Fig. S1).

Different from Yb- and Nd- based nanocrystals excitable only at 980 and 808 nm, respectively, Er-based nanocrystals can be efficiently excited at 980 and 1532 nm that is sought to optimal consideration of detection sensitivity, light penetration, and photothermal effects in the context of *in vivo* imaging^34^. During the time our manuscript was under review, additional papers have been published on the so-called new type of Er sensitized upconversion nanoparticles exploiting the upconversion excitation wavelength at 1500 nm^35–37^. Liu *et al*.^35^ designed a strategy for up-conversion core-shell nanoparticles using Er (1530 nm) as sensitizer, and Er (980 nm), Ho (1180 nm) and Nd (1060 nm) as activators in the second biological window. Also, they demonstrated the use of such nanoparticles for *in vivo* dynamic inflammation by use of a microneedle patch sensing at very high resolution (200 × 200 *μm*). Wang *et al*.^36^ developed Er enriched core–shell nanocrystals of NaErF_4_:20%Yb@NaLuF_4_ with efficient luminescence at 1525 nm under biocompatible excitation wavelength of 808 nm for bioimaging. Cheng *et al*.^37^ demonstrated a new type of up-conversion nanoparticle, sensitized by Er ions and extended the excitation wavelength to 1532 nm. Upon excitation, Er ions can transfer the absorbed energy to codoped activators, resulting in upconversion emission ranging from 400 to 1200 nm. Extending the excitation range of upconversion nanocrystals outside 800–1000 nm corresponding to traditional Nd and Yb sensitizers represents therefore an active and formidable challenge targeting applications such as anticounterfeiting, bioimaging, display, photovoltaics and information storage^35–39^.

Herein, we report a first and comprehensive description of the up-conversion emission properties of Er-Lu_2_O_3_ nanoparticles under ns pulsed excitation around 1500 nm monitored between 500 to 1100 nm. We highlight here the advantage offered by simultaneous use of pulsed laser excitation and time-gated luminescence detection. As such, nanoparticles that exhibit long lifetimes (from µs to ms range) in NIR biological window allow an improved contrast visualization^40^ by avoiding the tissue autofluorescence that is extended even in near infrared (>1000 nm^17^). The composition of nanoparticles was optimized by varying the concentrations of both Er activator and optically inert Li. Upconversion emission and indirect/direct decay measurements were performed considering multiple absorption transitions in the visible and near-infrared with specific aim to assess the effect of Li on the emission properties and to elucidate the up-conversion mechanisms. Besides optical excitation, we use low energy X-ray excitation and report the induced emission in an extended range ranging from 400 up to 1700 nm. The present results also reinforce our previous findings with Ln (Ln = Eu, Sm, Tb, Dy and Er) doped Y_2_O_3_ that established the role Li as a crystallization enhancer and not as local structure modifier^25^. A comparison between the optical and X-ray induced emission of Er-Lu_2_O_3_ and Er-Y_2_O_3_ nanoparticles is also discussed.

## Results and Discussion

### Summary of structural properties of Er-Lu_2_O_3_ nanoparticles

Several synthesis methods have been carefully considered to obtain the doped nanoparticles: hydrothermal synthesis, microemulsion reaction and the citrate complexation method. We disregarded the hydrothermal route due its low reproducibility of the final products and their dependence on the experimental conditions^41^. Further, the oil in water microemulsion reaction was disregarded due to the product separation process, the numerous washing steps as well as the highly dependence on the oil phase used^42^. We have selected the sol-gel citrate method^25^ due to the simplicity of the reaction steps and its excellent reproducibility, which is crucial for a study that compares nanoparticles of different (isostructural) hosts doped with Er and Li in varying concentrations as we propose here. At this stage, the advantage of excellent reproducibility was preferred against the disadvantage of agglomeration effect. Through the text, Er (1 and 7%) and Er(1 and 7%) Li (5%) (co)-doped Lu_2_O_3_ calcined at 800 °C are denoted as 1Er-Lu_2_O_3_ −800 °C, 7Er-Lu_2_O_3_ −800 °C, 1Er, 5Li-Lu_2_O_3_ −800 °C and 7Er, 5Li-Lu_2_O_3_ −800 °C, respectively. The calcination temperature of 800 °C for Li co-doped samples was imposed by elimination of unwanted lithium nitrate and Li volatility according to literature^25,43,44^. To compare the effect of improved crystallization triggered by thermal annealing to that induced by Li addition, Li free samples were also calcined at 1000 °C and denoted as reference samples.

**Figure 1:**
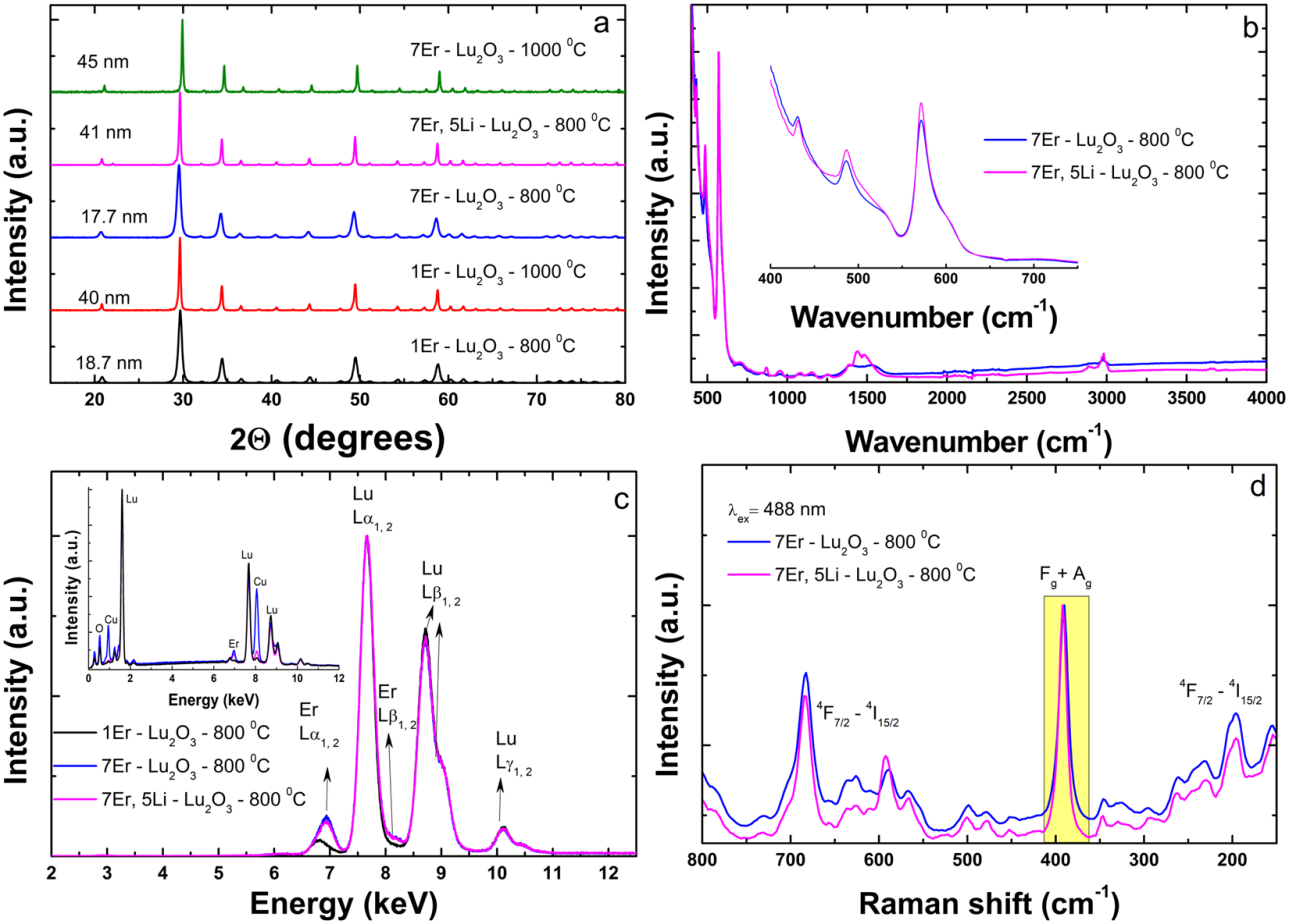
XRD patterns (**a**), FTIR (**b**), XRF (Inset: EDX spectra) (**c**) and Raman spectra excited at 488 nm (**d**) of Er-Lu_2_O_3_ nanoparticles. Highlighted with yellow in (**d**) is Lu_2_O_3_ phonon band.

Figure 1a illustrates the X-ray diffraction (XRD) patterns of Er(Li)-Lu_2_O_3_ nanoparticles that agree with those of cubic Lu_2_O_3_ (JCPDS card 86–2475). No additional peaks of other phases have been found, indicating the formation of a homogenous solid solution. The average crystallite sizes of samples calcined at 800 °C were estimated by use of Scherrer equation at around 17–19 nm for 1Er-Lu_2_O_3_ and 7Er-Lu_2_O_3_ and 41 nm for 7Er, 5Li-Lu_2_O_3_. Further calcination at 1000 °C increased the crystallite sizes of 1Er-Lu_2_O_3_ and 7Er-Lu_2_O_3_ from 17–19 to 40–45 nm.

Figure 1b depicts the Fourier transform infrared (FTIR) spectra of 7Er-Lu_2_O_3_ calcined at 800 °C and 7Er, 5Li-Lu_2_O_3_ −800 °C. The spectra are dominated by the absorption peaks at around 410, 490 and 580 cm^−1^ due to Lu–O bond vibration in Lu_2_O_3_^45^. It is worth noting that only feeble bands were detected at ~ 1630 cm^−1^ (deformation vibration) or in the 3000–3600 cm^−1^ (stretching vibration) region corresponding to O-H bond, indicating the absence of coordinated or adsorbed water, respectively. X-ray fluorescence (XRF) spectra in Fig. 1c and Energy Dispersive X-ray (EDX) (inset of Fig. 1c) spectra evidences the bulk elemental composition given by Lu and Er metals in agreement with the nominal composition without interference from impurity metals. The Raman spectra show the presence of the F_g_ + A_g_ vibration mode of the Lu_2_O_3_ (393 cm^−1^)^1^. The phonon bands are buried into much more intense f-f luminescence of Er corresponding to ^4^F_7/2_-^4^I_15/2_ emission transition induced by laser excitation at 488 nm.

**Figure 2:**
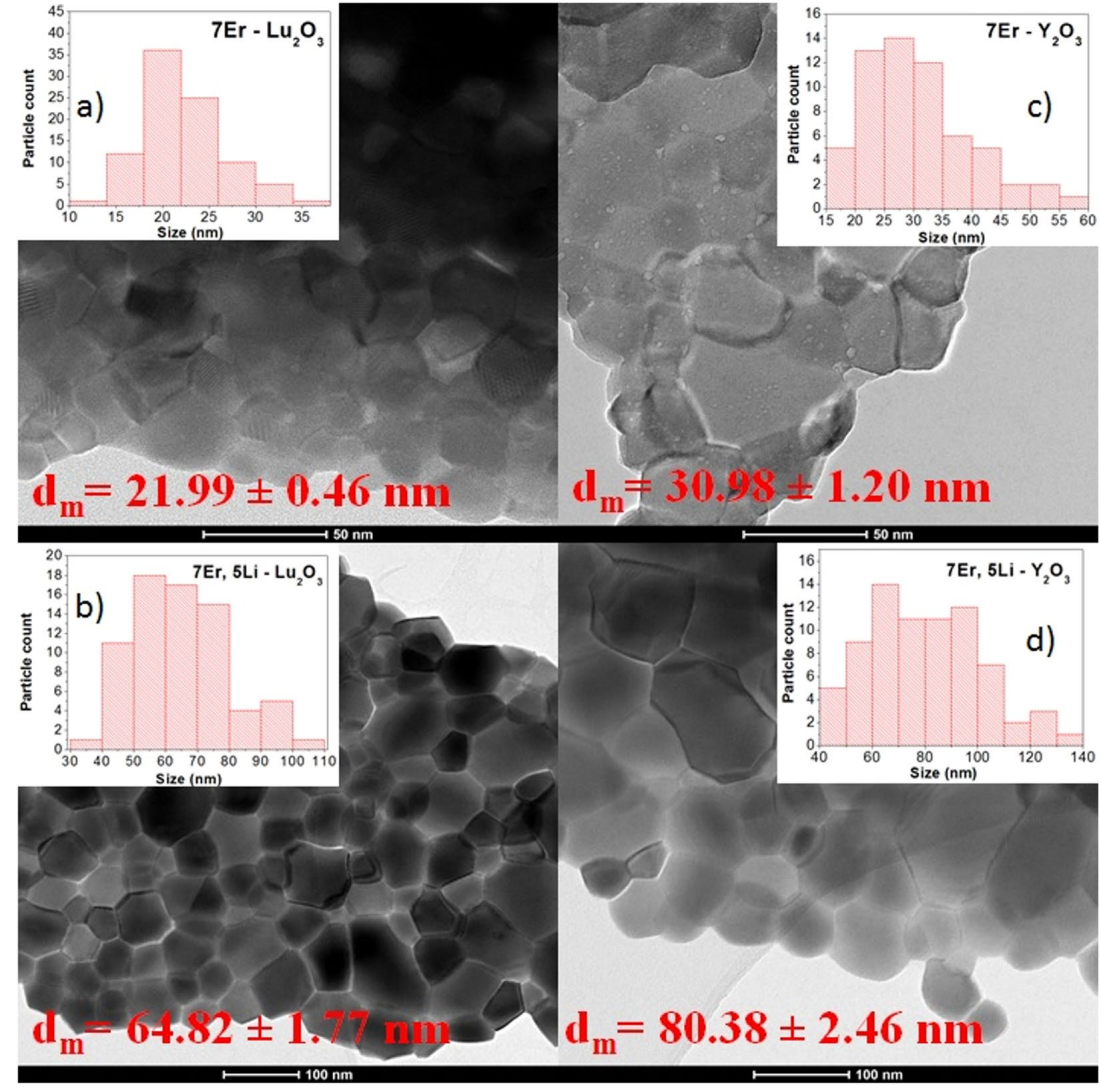
TEM Micrographs obtained on (**a**) 7Er-Lu_2_O_3_ −800 °C, (**b**) 7Er, 5Li-Lu_2_O_3_ −800 °C, (**c**) 7Er-Y_2_O_3_ −800 °C and (**d**) 7Er, 5Li-Y_2_O_3_ −800 °C nanoparticles.

By analyzing the TEM data presented in Fig. 2, we can state that the powder consists from polyhedral nanoparticles that are forming mild to hard agglomerates. The average particle size is ranging from ~22 nm for 7Er-Lu_2_O_3_ −800 °C, in agreement with X-ray diffraction data, while the Li addition induces the increase of the particle size to ~ 65 nm, higher than the value obtained from X-ray data calculated using Scherer equation as particle seem to be composed of two-three crystallites. For comparison, in Fig. 2 are presented the TEM images for its isostructural counterpart, Y_2_O_3_ powder. In this case, the average particle sizes are slightly higher than those of Lu_2_O_3_, ranging from ~31 nm for 7Er-Y_2_O_3_ −800 °C to ~80 nm for 7Er, 5Li-Y_2_O_3_ −800 °C nanoparticles.

Carlos *et al*.^46^ extensively studied the effect of co-doping Er, Yb-Y_2_O_3_ with Li on the nanoparticle structure by use of X-ray diffraction and Rietveld refinement as well as TEM analysis, suggesting that Li additions shrinks the lattice constant and increases the nanoparticle size without substituting the Y cation, probably residing interstitially. We observe the same trend here, that is, the lattice constant shrinks from 10.48 (7Er-Lu_2_O_3_ −800 °C) to 10.44 Å (7Er, 5Li-Lu_2_O_3_ −800 °C) while the nanoparticles size increases from 22 nm to 65 nm (Fig. 2).

### Up-conversion of Er-Lu_2_O_3_ nanoparticles upon excitation around 1500 nm

#### Composition optimization

The up-conversion brightness of Er based materials is closely related to the balance between the absorption of the incident light and the quantum efficiency^47^. The up-conversion quantum efficiency is direct related with Er-Er distance *via* Er concentration and host selection/manipulation^48,49^. The probability of energy transfer in Er-Er pairs considering the most common dipole-dipole mechanism can be written as ${\rho }_{Er-Er}=\frac{1}{{\tau }_{Er}}{(\frac{{R}_{0}}{R})}^{6}$where *τ*_*Er*_ is the actual lifetime of the Er excited state, including multiphonon radiative decay, and *R*_0_ is the critical transfer distance for which excitation transfer and spontaneous deactivation of the Er dopant have equal probability^47^. We assume that Er doping does not affect the lattice parameter of Lu_2_O_3_ and use thus as lattice parameter, a, the value of 10.48 Å for 7Er and 10.43 Å for 1Er (calculated from XRD data, Fig. 1a). Lu_2_O_3_ crystallizes in a space group in the *C*-type metal oxide structure, $Ia\bar{3}$ space group and its unit cell correspond to the structured cell formula Lu_32_O_48_. This means that there is Z = 32 Lu ions in each unit cell. The distribution of Er dopant is considered to be randomly on the low symmetry, C_2_ and high-symmetry, S_6_/C_3i_ sites with a relative contributions of 3:1^4^. The average Er-Er distance *R*, can be approximated: ${R}_{L{u}_{2}{O}_{3}}={(\frac{{a}^{3}\sqrt{3}/2}{32x})}^{\frac{1}{3}}$, where *x* is the Er doping percentage. According to this equation, the average Er-Er in distance in 1Er and 7Er-Lu_2_O_3_ samples can be estimated around 16.39 and 8.57 Å, respectively. The optimum average Er-Er distance obtained for the most emissive sample in Y_2_O_3_, 7%Er^25^ was estimated at 8.67 Å (using 10.602 Å as lattice parameter^4^). The Er-Er distance obtained in 7Er-Lu_2_O_3_ nanoparticles is only 2% smaller than obtained for its Y_2_O_3_ homologue. Therefore, we assume a similar optimum concentration of Er of 7% in Lu_2_O_3_ further sustained by preliminary measurements on varying Er concentrations. The values of Er-Er distances for the optimum upconversion intensity come close to those reported for β-NaYF_4_ and Gd_2_O_2_S hosts with optimum average Er−Er distances of 8.8 and 8.3 Å, respectively^50^.

Another well-known strategy to enhance the up-conversion emission intensity is by host lattice manipulation, that is, modification of the local structure around lanthanide dopants by co-doping with optically inert ions, such as monovalent Li^43,44,49,51–54^, monovalent K^55^, divalent Zn^56^, Cu^57^ or Mg^58,59^. Li is the smallest metallic ion in the periodic table with an ionic radius of 0.9 Å. As such, Li is considered to be easily incorporated into various host lattices, including oxides^49^. Carlos *et al*.^46^ reports an 10 times increase of the upconversion quantum yield when adding Li on Y_2_O_3_:Yb, Er nanoparticles mainly due to the particle size increment and lattice parameter decrease. Also, they indicated the number of OH^−^ groups does not change significantly with increasing Li content while the role of carbonate groups as upconversion emission quencher was ruled out. This report confirm our previous findings, that is, Li enhances the upconversion emission by improving the crystallization rather than distorting the local structure around the activator^25^.

#### Overview of the up-conversion emission spectra

**Figure 3:**
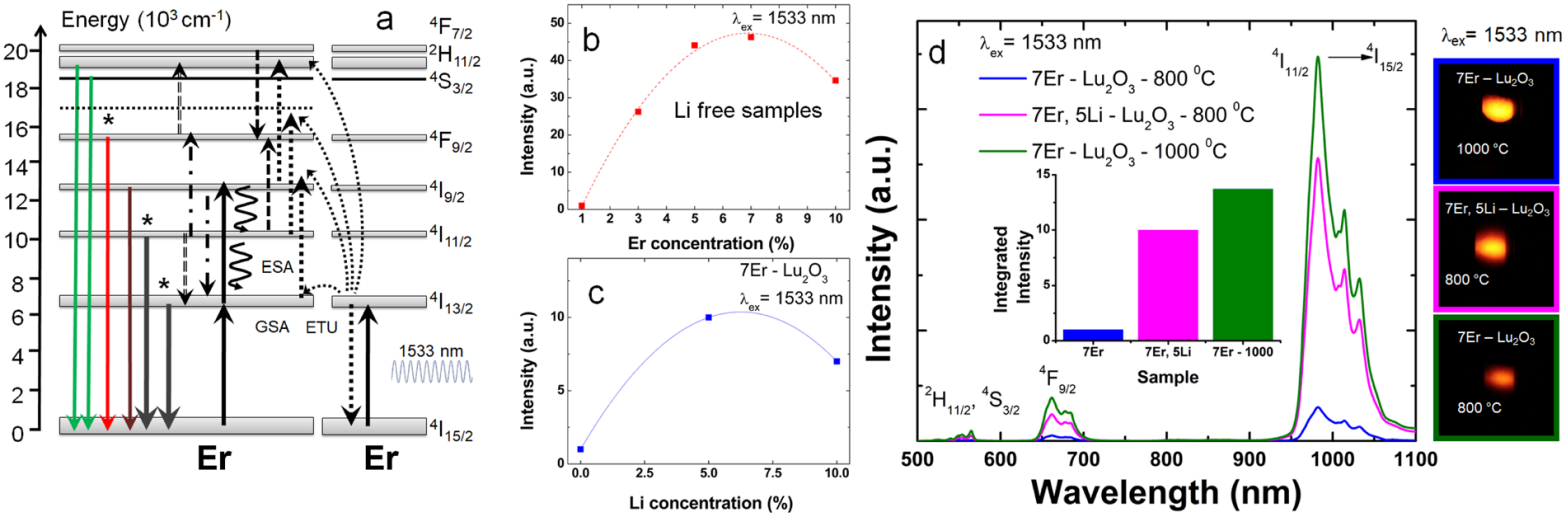
(**a**) Schematic representation of the relevant absorption and emission transitions and the possible mechanisms responsible for the up-conversion emission of Er-Lu_2_O_3_ following excitation at 1533 nm. Curved arrows indicate multiphonon relaxation; coloured lines correspond to the emission transitions and dashed lines indicate the non-radiative energy transfer/cross relaxation processes. With “*” are labelled the energy levels selected in measurement of the emission decays. Dependence of up-conversion emission intensity of Er-Lu_2_O_3_ upon 1533 nm excitation with Er (**b**) and Li (**c**) concentration (**d**) Up-conversion emission spectra of 7Er-Lu_2_O_3_ −800 °C, 7Er, 5Li-Lu_2_O_3_ −800 °C and, for comparison, 7Er-Lu_2_O_3_ −1000 °C (reference sample). All samples were placed in a standard sample holder (14 mm × 7 mm). The digital images were obtain in the *dark room light conditions* by use of Canon EOS 60D under exposure time of 1 s with 400 ISO. The energy of the excitation is around 1.85 mJ (average power density of ~65 mW/cm^2^).

Figure 3a illustrates the main emission/excitation transitions of Er-Lu_2_O_3_ under ~1500 nm excitation. The dependence of the up-conversion emission intensity under 1533 nm excitation with Er concentration (1–10%) and Li addition (0, 5 and 10%) is presented in Fig. 3b,c. The optimum composition yielding the highest up-conversion intensity is 7% and 5% for Er and Li concentration, respectively. Figure 3d gathers the up-conversion emission spectra of optimized 7Er-Lu_2_O_3_ series: 7Er-Lu_2_O_3_ −800 °C; 7Er, 5Li-Lu_2_O_3_ −800 °C and 7Er-Lu_2_O_3_ −1000 °C, the reference sample. Upon ~1500 nm excitation, Er display typical emission bands assigned to ^2^H_11/2_, ^4^S_3/2_–^4^I_15/2_ (green emission); ^4^F_9/2_–^4^I_15/2_ (red emission); ^4^I_9/2_–^4^I_15/2_ and ^2^H_11/2_, ^4^S_3/2_–^4^I_13/2_ (790–850 nm) and ^4^I_11/2_-^4^I_15/2_ (980 nm) emission transitions. The 980 nm based emission represents 89–93% (compared to almost 99% for 1Er-Lu_2_O_3_; data not presented) of the total up-conversion emission as observed previously for its Y_2_O_3_ counterpart^25^.

As shown in Fig. 3d codoping with Li or increasing the anneal temperature to 1000 °C induce a similar increase by a factor of 10–13 of the up-conversion emission intensity. Since both samples present a similar crystallite sizes around 40–45 nm, such increase of up-conversion emission intensity by Li addition is explained by improved crystallization. Moreover, more than 40% of the 980 emission intensity falls above 1000 nm, outside the water absorption biological media^17^. To the best of our knowledge, less than 10 studies report so far in the literature on (almost) pure/monochromatic up-conversion emission around 980 nm under up-conversion excitation in Er(Yb) based nanosized systems.

Finally, taking advantage of our OPO tunable excitation laser, we compare the up-conversion emission shapes defined by red to green emission ratio (RGR)^60^, measured under 1533 and 980 nm excitation. As shown in Fig. S2, the two types of emission spectra display rather close colors, reddish yellow (excitation at 1533 nm) and yellowish orange (excitation at 980 nm) likely due to similar up-conversion energy transfer mechanisms responsible for populating the green and red emission levels (^2^H_11/2_, ^4^S_3/2_ and ^4^F_9/2_, respectively) for 7Er-Lu_2_O_3_ nanoparticles.

#### Up-conversion mechanisms

As proposed by Auzel^47^, the two main up-conversion mechanisms are ground state absorptions followed by excited state absorptions (GSA/ESA) and energy transfer up-conversion mechanism (ETU) (see also Fig. 3a). To characterize the up-conversion mechanisms under 1533 nm excitation, we have measured (i) dependence of up-conversion emission intensity on the laser pulse energy, (ii) up-conversion emission decays and comparison with down-conversion (indirect and direct excitations) and (iii) up-conversion excitation spectra.

**Figure 4:**
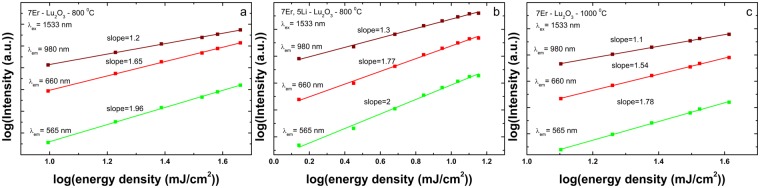
The dependence of up-conversion emission intensity monitored at 565, 675 and 980 nm on the laser pulse energy upon 1533 nm excitation in log-log scale illustrated for 7Er-Lu_2_O_3_ −800 °C (**a**); 7Er, 5Li-Lu_2_O_3_ −800 °C (**b**) and, for comparison, 7Er-Lu_2_O_3_ −1000 °C (reference sample) (**c**).

Dependence of up-conversion emission intensity on the laser pulse energy: As shown in Fig. 4, slope values of 1.8–2 (^2^H_11/2_, ^4^S_3/2_ emission monitored at 565 nm) or 1.54–1.77 (^4^F_9/2_ emission monitored at 675 nm) and 1.1–1.3 (^4^I_11/2_ emission monitored at 980 nm) were estimated for 7Er-Lu_2_O_3_–800 °C; 7Er, 5Li-Lu_2_O_3_ −800 °C and reference sample (7Er-Lu_2_O_3_ −1000 °C) suggesting at first glance a one and two photon process for these transitions^61^. All values are below the theoretical values of 3 and 2, respectively, due to the high-power density excitation^29,62^ and the influence of up-conversion over linear decay for the depletion of corresponding intermediate excited states^63^. For high power density, a slope of one is expected for a two-photon ETU process^62^. This reduction of the slope confirms that our experimental conditions correspond to a high-power regime (~10^6^ W/cm^2^) *during* the excitation pulses (5 ns). Therefore, due to the saturation of the dependence of the up-conversion emission intensity on the laser pulse energy in high-power regime, we cannot depict the true number of photons needed to populate the emitting levels. A similar behavior was observed by Martín-Rodríguez *et al*.^29^ for Er doped Gd_2_O_2_S under similar excitation conditions using an OPO laser system with 2 mJ pulse energy for 10 ns pulse width and 20 Hz repetition rate. Using a cw high power excitation source, Pollnau *et al*.^61^ and Suvyer *et al*.^62^ observed saturation of the dependence of the up-conversion emission intensity on the laser pump power. Finally, the dependencies in Fig. 4a,b show that addition of Li does not change the mechanisms of UPC emission established in Li free sample.

**Figure 5:**
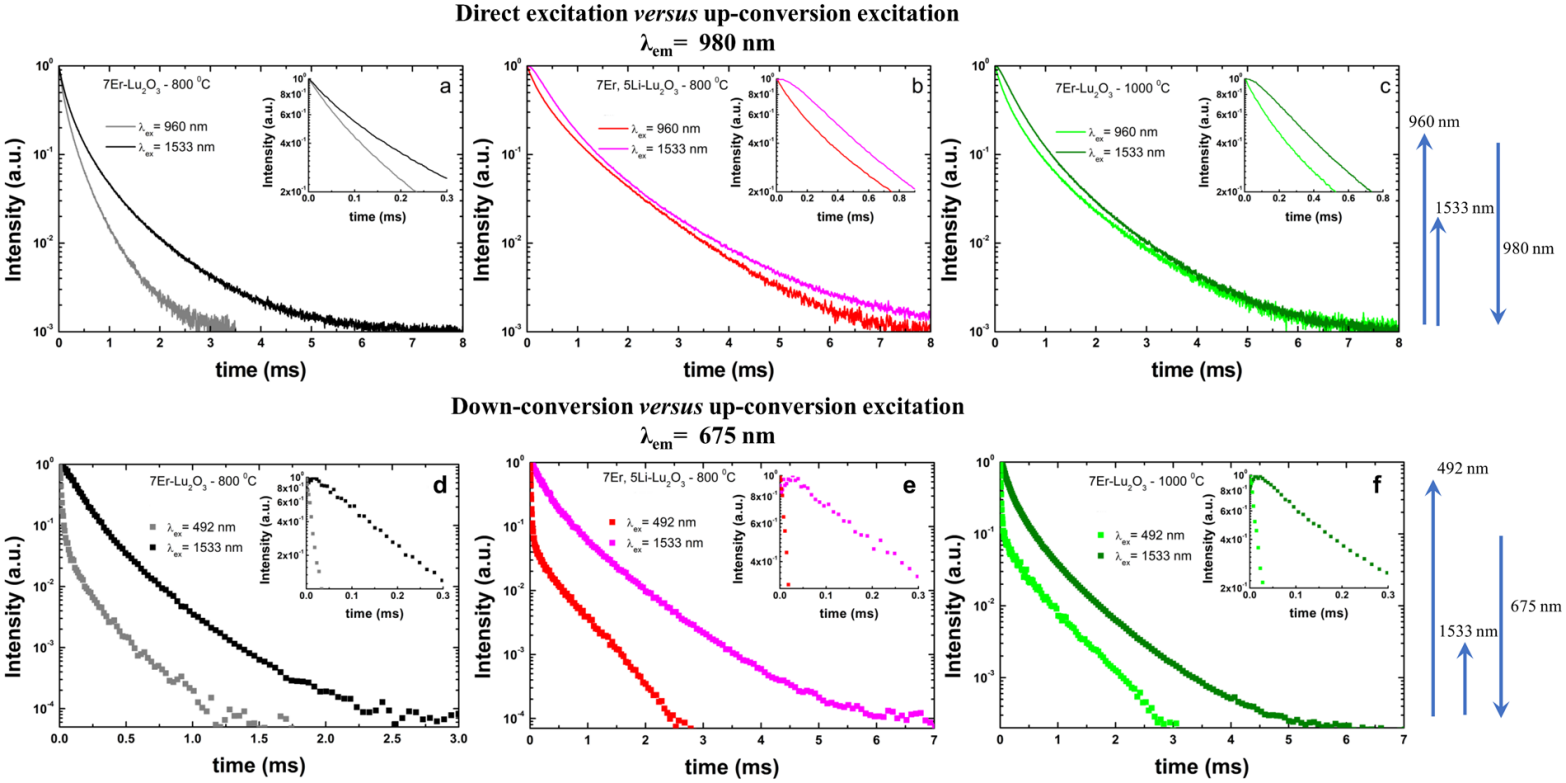
Comparison between the direct/down- and up-conversion emission decays of 7Er-Lu_2_O_3_ −800 °C (**a**,**d**); 7Er, 5Li-Lu_2_O_3_ −800 °C (**b**,**e**) and, for comparison, 7Er-Lu_2_O_3_ −1000 °C (reference sample) (**c**,**f**). (**a**–**c**) The emission was monitored around 980 nm under direct (960 nm) and up-conversion excitation (1533 nm). (**d**–**f**) The emission was monitored around 675 nm under down- (492 nm) and up-conversion excitation (1533 nm).

Up-conversion emission decays: It is well-known that, using a pulsed excitation the GSA/ESA and ETU mechanisms can be readily differentiated by comparing the emission decays measured upon down- and up-conversion excitations^47^. Up-conversion by energy transfer mechanism typically leads to a decay curve for the up-conversion emission that is longer than that measured in down-conversion or direct excitation mode since the emission level is fed continuously after the excitation pulse by a long-lived intermediate state population (e.g. ^4^I_13/2_) that is also decaying^47,64^. Figure 5 presents the comparison of emission decays corresponding ^4^I_11/2_ (NIR emission) and ^4^F_9/2_ (red emission) level for 7Er-Lu_2_O_3_ −800 °C; 7Er, 5Li-Lu_2_O_3_ −800 °C and, for comparison, 7Er-Lu_2_O_3_ −1000 °C under direct/down- and up-conversion excitations at 960/492 nm and 1533 nm, respectively.

The lengthening of the NIR emission decays under up-conversion compared to down-direct excitation (see also Figure 5 and Table 1) confirms the presence of energy transfer mechanisms that fuels the ^4^I_11/2_ level under up-conversion excitation at 1533 nm (see also Scheme from Fig. 3a) through long lived ^4^I_13/2_ energy level. Due to the power density of the excitation laser and looping energy transfer up-conversion mechanisms that affects the ^4^I_11/2_ emission^65^ (see also Scheme for Fig. 3a), the up-conversion emission decay is not greatly lengthened compared to the direct excitation emission decay (Fig. 5a–c and Table 1) as is the case of ^4^F_9/2_ up-conversion emission decays (Fig. 5d–f) while their respective rise times are of few microseconds. A similar effect was observed by Martín-Rodríguez *et al*. for Er doped Gd_2_O_2_S (microparticles)^29^ and by Chen *et al*. for Er doped LiYF_4_^33^. Also, for higher pump laser energy density regime (peak power >10^5^ W/cm^2^) the emission decay rise time shortens^29^ due to the saturation of the initial ground state absorption, leading to a maximum of the subsequent (^4^I_13/2_, ^4^I_13/2_) → (^4^I_15/2_, ^4^I_9/2_) energy transfer up-conversion rate.

**Table 1:** Average lifetimes of 7Er-Lu_2_O_3_ nanoparticles under down-conversion (λ_ex_ = 492 nm), direct excitation (960 nm) and up-conversion excitation (λ_ex_ = 1533 nm) monitoring the 675 and 980 nm based emissions.

λ_ex_/λ_em_ =	Estimated average lifetime (ms)			
	Down-conversion excitation	Direct excitation	Up-conversion excitation	
Sample	492/675 nm	960/980 nm	1533/675 nm	1533/980 nm
7Er-Lu_2_O_3_ −800 °C	0.019 ± 10^−3^	0.19 ± 5 × 10^−3^	0.154 ± 3 × 10^−3^	0.29 ± 5 × 10^−3^
7Er, 5Li-Lu_2_O_3_ −800 °C	0.035 ± 10^−3^	0.56 ± 5 × 10^−3^	0.331 ± 3 × 10^−3^	0.65 ± 5 × 10^−3^
7Er-Lu_2_O_3_ −1000 °C	0.052 ± 10^−3^	0.38 ± 5 × 10^−3^	0.299 ± 3 × 10^−3^	0.53 ± 5 × 10^−3^

*All values were estimated by integrating the area of the normalized decay (see Experimental Section).

**The rise time measured under 1533 nm excitation is only a few µs due to the efficient cross relaxation processes between Er ions.

It is further observed that upon Li addition we observe similar effects on up-conversion emission intensity and decay as increasing the calcination temperature from 800 to 1000 °C. As such, the up-conversion emission intensity is enhanced by a factor of 10–13 (Fig. 3c) and the average decay times corresponding to red (675 nm) and NIR emission (980 nm) transitions are enhanced by a factor of 2–3 (Table 1).

**Figure 6:**
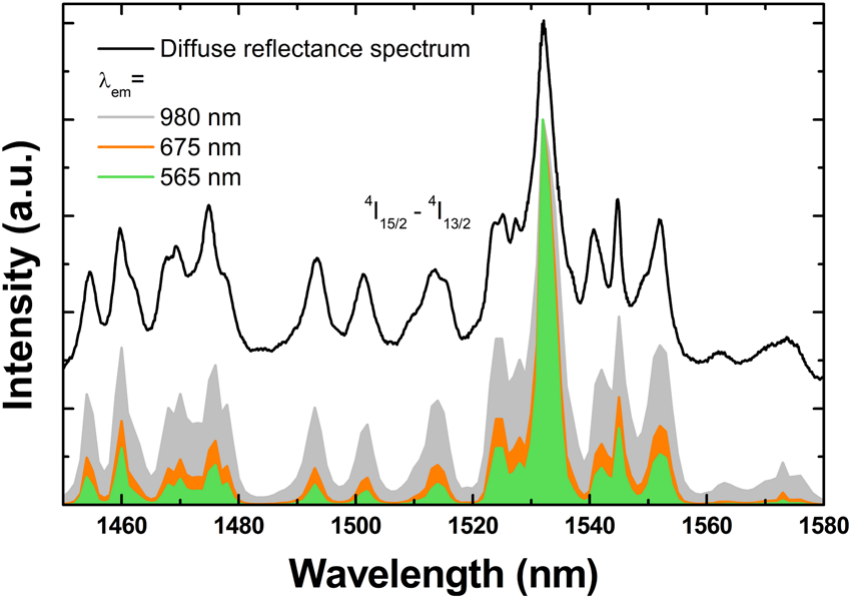
Comparison of the up-conversion excitation spectra of 7Er, 5Li-Lu_2_O_3_ −800 °C monitored at 565, 675 and 980 nm with diffuse reflectance spectrum. All excitation spectra were normalized at the maximum absorption intensity (1533 nm). Similar excitation spectra were recorded for 7Er-Lu_2_O_3_ −800 and 1000 °C.

Up-conversion excitation spectra: The comparison of the up-conversion excitation spectra of 7Er, 5Li-Lu_2_O_3_ −800 °C that monitor the emissions at 565, 675 and 980 nm is presented in Fig. 6. With increasing the monitored emission wavelength, the lines get narrower due to the greater number of photons required to populate the green and red emitting levels (theoretically three photons), whilst only two photons (theoretically value) are necessary for the ~980 nm emission corresponding to the ^4^I_11/2_-^4^I_15/2_ emission transition^22^. Altogether, the shapes of the up-conversion excitation spectra and their resemblance to the diffuse reflectance absorption spectrum (see the diffuse reflectance spectrum illustrated with black line in Fig. 6) from ground state (^4^I_15/2_) to initial excited state (^4^I_13/2_) confirm the predominance of ETU mechanism^47^. Because otherwise, new lines would emerge corresponding to the convolution of all involved intermediary states due to the GSA/ESA up-conversion mechanism processes^47^. As a Kramers lanthanide, the maximum theoretical number of lines corresponding to ^4^I_15/2_-^4^I_13/2_ transition of Er assuming that only the fundamental level is populated at room -temperature is 16. The number observed is definitely larger (including well resolved lines but also the so -called spectral shoulders) which means that (at least) the next level to the fundamental one is also populated.

Considering the dependence of up-conversion emission intensity on the laser pulse energy, up-conversion emission decays and up-conversion excitation spectra described above, we discuss the mechanisms responsible for the up-conversion emission as following (see also Fig. 3a). Upon excitation into the long – lived ^4^I_13/2_ level (~0.6 ms as measured for 7Er, 5Li-Lu_2_O_3_ −800 °C that is significantly shortened compared to 1Er-Lu_2_O_3_ −800 °C around 1.5 ms), Er can be excited to the ^4^I_9/2_ energy level which decays mostly non-radiatively on the ^4^I_11/2_ energy level that can lead to a strong 980 nm based up-conversion emission. From the excited ^4^I_11/2_ energy level, the ^4^F_9/2_ state is populated *via* the (^4^I_11/2_^4^, I_9/2_) → (^4^F_9/2_^4^, I_13/2_) energy transfer mechanism giving rise up-conversion emission in red spectral range *via* a three-photon process. Also, there are secondary cross relaxations energy transfer mechanisms that favors the ^4^I_13/2_ emission such as ^4^F_9/2_-H_11/2_^4^, S_3/2_ ↔ ^4^I_11/2_-^4^I_13/2_ and ^4^I_11/2_-^4^F_9/2_ ↔ ^4^I_9/2_-^4^I_13/2_ as observed in Fig. S3 when increasing the Er concentration from 1% to 7%. The lengthening of the up-conversion emission decays compared to down-conversion emission decays (Fig. 5 and Table 1) is a clear indicator of the efficient contribution of up-conversion mechanisms to the overall emission^47^ besides GSA/ESA mechanisms as confirmed further by the analysis of up-conversion excitation spectra (Fig. 6).

Compared to Y_2_O_3_, Lu_2_O_3_ has a smaller lattice parameter (10.393 Å compared to 10.604 Å), that will lead to slightly different crystal field and thus to a spectral shift of their emission spectra^8^. Although the Er-Y_2_O_3_ and Er-Lu_2_O_3_ nanoparticles were synthesized by using the same procedure, the resulted nanoparticles are of distinct size (Fig. 2), Lu_2_O_3_ being smaller (~22 nm) than Y_2_O_3_ (~31 nm) in size for the same Er concentration (7%) and thermal treatment (800 °C). This indicates that the mechanism of nucleation and growth of nanoparticles in solution is different for the two oxides having consequently the formation of crystallite with different sizes. This phenomenon can be described through the La Mer burst nucleation^66,67^ that depends strongly on the reaction conditions: even a slight change in condition, such as pH or solution concentration, can lead to a completely different mechanism. For example, in the Ostwald ripening process for growing, due to the high solubility and the surface energy of smaller particles within solution, these redissolve and in turn allow the larger particles to grow even more; higher is the surface energy, bigger crystallite will be formed. This could be one explanation for the different crystallite size, due to the fact that, indeed, the two hydroxides present different free Gibbs energies^68^. Analyzing the effect of Li addition, the effect is similar for both powders. Thus, for the Lu_2_O_3_ nanoparticles, the Li addition induces a particle growth by 2.9 times while in the case of Y_2_O_3_ induces a growth of 2.6 times. Another effect, in what concerns the nanoparticle size distribution in the case of Li addition is that it induces a tendency of bimodal distribution of nanoparticles, as we can see in the insets of each sample in Fig. 2. The increase of crystallinity leads to a different emission lifetime, the greater the nanoparticle, the lower the surface to volume ratio leading to longer emission decay^69^. Differences exist also in emission decays monitoring the 980 and 1533 nm under direct excitation at 960 and 1470 nm, respectively (Fig. S4). In this case, although the average lifetimes are roughly similar, the decay shapes are obviously different, suggesting that a different balance of radiative and non-radiative processes govern the Er emission in the two isostructural hosts. Finally, the comparison of the up-conversion emission intensity of 7Er(5Li)-Lu_2_O_3_ −800 °C and 7Er(5Li)-Y_2_O_3_ −800 °C measured under the same experimental conditions under 1533 nm excitation, reveal that Y_2_O_3_ renders brighter up-conversion emission by a factor of 1.3 for 7Er, 5Li and a factor of 4.7 for 7Er samples (Fig. S5). Similar trends were measured using 980 nm as up-conversion excitation wavelength (using both pulsed OPO and cw laser diode). This observation contradicts the two order of magnitude enhancement observed for 1%Er-Lu_2_O_3_ relative to 1%Er-Y_2_O_3_ under 980 nm cw excitation^3^

### X-ray induced emission on Er-Lu_2_O_3_ nanoparticles

We further present the emission properties of Er-Lu_2_O_3_ nanoparticles under X-ray excitation measured from visible to NIR by use of X-ray source with energy and flux in the medical diagnostic range (Mo target, up to 2 Gy/s). According to literature, the X-ray induced emission intensity of Eu/Tb doped Lu_2_O_3_ and commercially available Eu/Tb: Gd_2_O_2_S^70–72^ and CsI:Tl^70^ and CaWO_4_^73^ are comparable, or even brighter for some X-ray energies (such as: 50–100 kVp for Lu_2_O_3_:Eu compared to Gd_2_O_2_S:Eu^71^). Eu doped Lu_2_O_3_ was reported to be a suitable candidate for X-ray tomography screens as thin film^73,74^, ceramics^70^ and nanopowder^71,72^. However, to the best of our knowledge, no reports on X-ray induced emission for Er-doped Lu_2_O_3_ were found.

**Figure 7:**
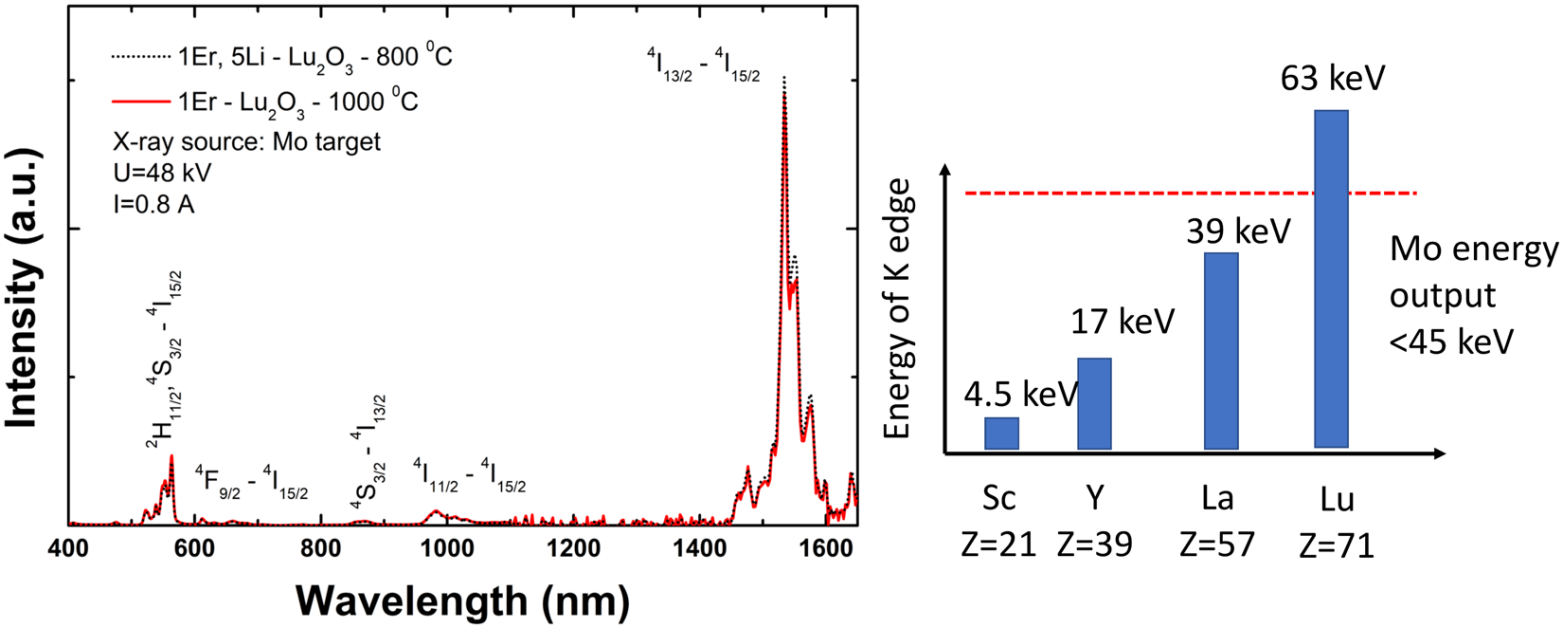
(left) X-ray induced luminescence emission spectra of 1Er, 5Li-Lu_2_O_3_ −800 °C and, for comparison, 1Er-Lu_2_O_3_ −1000 °C (reference sample) in the extended range (400–1700 nm). (right) Schematic representation of the comparison of Y and Lu K edge in respect to the Mo X-ray source energy output.

Figure 7 shows the X-ray induced luminescence spectra of the most emissive samples, 1Er, 5Li-Lu_2_O_3_ −800 °C and, for comparison, 1Er-Lu_2_O_3_ −1000 °C in the extended range from UV to NIR (400–1700 nm). Under X-ray radiation, Er luminescence is achieved *via* three main processes: (i) the absorption of high energy photons, (ii) generation of electron -hole pairs and (iii) the recombination of some of these pairs leading to energy transfer to Er^75^. Thus, under X-ray radiation, Er exhibits emission from UV to NIR region as follow: 410 nm (^2^H_9/2_–^4^I_15/2_); 480 nm (^4^F_7/2_–^4^I_15/2_); 565 nm (^2^H_11/2_, ^4^S_3/2_–^4^I_15/2_); 660 nm (^4^F_9/2_–^4^I_15/2_); 870 nm (^4^S_3/2_–^4^I_13/2_), 980 nm (^4^I_11/2_-^4^I_15/2_) and 1500 nm (^4^I_13/2_-^4^I_15/2_). As previously observed for Y_2_O_3_ nanoparticles^25^, the strongest emission under X-ray radiation is obtained with Er concentration of 1% preserving the characteristic emission spectrum.

According to Fig. 7, the X-ray induced luminescence of 1Er-Lu_2_O_3_ −1000 °C is dominated by the NIR emission at 1500 nm (^4^I_13/2_-^4^I_15/2_) representing ~80% of the total emission intensity.

**Figure 8:**
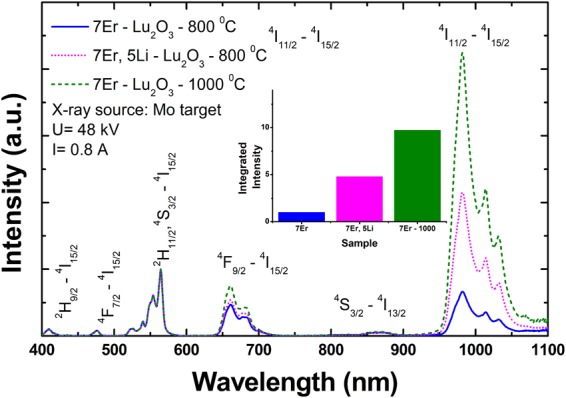
Comparison of X-ray induced luminescence emission spectra for 7Er-Lu_2_O_3_ −800 °C; 7Er, 5Li-Lu_2_O_3_ −800 °C and, for comparison, 7Er-Lu_2_O_3_ −1000 °C (reference sample) measured in the 400 to 1100 nm range. The spectra are normalized at 565 nm for comparison purposes. The inset compares the integrated emission intensity of 7Er-Lu_2_O_3_ −800 °C; 7Er, 5Li-Lu_2_O_3_ −800 °C and 7Er-Lu_2_O_3_ −1000 °C (reference sample). The emission in the near infrared range (1100–1700 nm) has a very low signal to noise ratio (is too weak to be clearly observed) and is not represented.

To assess the changes in the emission shape and intensity of 7Er-Lu_2_O_3_ nanoparticles under X-ray excitation induced by Li co-doping, we further measured the X-ray induced luminescence of 7Er-Lu_2_O_3_ −800 °C; 7Er, 5Li-Lu_2_O_3_ −800 °C and for comparison, 7Er-Lu_2_O_3_ −1000 °C from 400 to 1100 nm range (Fig. 8).

The red to green ratio changes from 1.2 for 7Er(5Li)-Lu_2_O_3_ −800 °C to 1.65 for 7Er-Lu_2_O_3_ −1000 °C and the near infrared to green ratio, from 2.7 for 7Er-Lu_2_O_3_ −800 °C to 3.43 for 7Er, 5Li-Lu_2_O_3_ −800 °C and to 8 for 7Er-Lu_2_O_3_ −1000 °C. The 980 nm emission represents 47% of the total emission for 7Er-Lu_2_O_3_ −800 °C and increases up to 80% for 7Er-Lu_2_O_3_ −1000 °C. The integrated luminescence emission increases by a factor of 5 and 10 with Li addition and extending the anneal temperature up to 1000 °C, respectively (Fig. 8). As shown in Fig. 8, the X-ray induced luminescence of Er is highly similar to optical induced emission (Fig. S6).

As concerns the comparison of X-ray induced emission properties of Er-Lu_2_O_3_ and Er-Y_2_O_3_, we note that Y K-edge is 17.0 keV, within the diagnostic range (<45 keV) of the Mo source, while the Lu K-edge at 63.3 keV falls outside the energy range of the Mo target (Scheme in Fig. 7). Still, the X-ray induced emissions are comparable in intensity likely due to the higher atomic number of Lu (Z = 71) compared with Y (Z = 39). The upconversion emission properties are expected to be similar across cubic sesquioxides due to the same physical-chemical properties: maximum phonon band (F_g_ + A_g_ maximum phonon band at around 400 cm^−1^ and cut-off phonon at <600 cm^−1^^1^), same random distribution in C_2_ and C_3i_, same local environment/crystal-field. In contrast, the X-ray luminescence intensity is dependent on the cations K-edge (Sc −4.486 keV; La −38.894 keV; Pr −41.958 keV; Nd −43.538 keV and from Sm −46.801 keV to Lu −63.311 keV) and X-ray energy. X-ray activated near-infrared luminescence benefits from deep penetration in both excitation (Mo X-ray source, less than 5 cm penetration depth^76^) and emission (around 1 cm penetration depth for 1500–1550 nm)^17^. Keeping in mind the obvious advantages offered for deep penetration bio-imaging applications, targeted studies using X-ray induced NIR emission in biocompatible nanoparticles are emerging (e.g.^77,78^).

## Conclusions

In summary, we have investigated the properties of Er-Lu_2_O_3_ nanoparticles synthesized by citrate complexation method under near-infrared up-conversion (1500 nm) and X-ray excitations in the 400 to 1700 nm range. The composition was varied in terms of Er concentration (1 to 7%) and Li addition (5 and 10%), being optimum for 7%Er and 5% Li. Upconversion emission and indirect/direct decay measurements were performed considering multiple absorption transitions in the visible and near-infrared with specific aim to assess the effect of Li on the emission properties and to elucidate the up-conversion mechanisms. Under 1500 nm excitation, the nanoparticles present an almost near infrared monochromatic up-conversion emission centered at 980 nm while over 80% of the X-ray induced emission is concentrated around 1500 nm. Li enhancement of up conversion emission (up to one order of magnitude) is related to improved crystallization which confirm our previous findings and recently published literature. Although the comparison between the emission properties of Er-Lu_2_O_3_ and Er-Y_2_O_3_ nanoparticles of similar composition, synthesized by a similar method and measured under similar experimental conditions is not straightforward, we evidence that Lu_2_O_3_ is not exceeding the efficiency of Y_2_O_3_ as upconversion host as commonly sought. We believe that use of Er sensitized (under 1500 nm excitation) emission of near infrared emissions of upconversion activators (Er at 980 nm, Ho at 1200 nm and Nd at 1060 nm) in optimized cubic sesquioxides will lead to the development of novel near-infrared nanoprobes as recently anticipated in the literature.

## Materials and Methods

### Synthesis

Er(1–10%) and Er(1 and 7%) Li (5 and 10%) (co)-doped Lu_2_O_3_ were prepared using the citrate complexation method described elsewhere^79^. The Er, Li-Lu_2_O_3_ samples were calcined in air at 800 °C for 4 hours at a heating rate of 5 °C/min to ensure the complete elimination of the unwanted nitrate species which vaporizes at 600 °C (CSID:8305408). Also, this temperature was chosen for Li containing samples, because of the Li component which is volatile at elevated temperature. Therefore, only the Li free, Er-Lu_2_O_3_ were also calcined at 1000 °C for 4 hours to follow the influence of the crystallite size and compare it with Li addition.

### Characterization

Powder X-ray diffraction (XRD) patterns were recorded on a Bruker-AXS D8 Advance diffractometer equipped with a one-dimensional detector (LynxEye type) using Cu-Kα radiation (0.154178 nm) at a scanning speed of 0.10 degrees min^−1^ in the 15–90 degrees 2θ range. Lattice parameters are calculated using the Bragg’s law. The following equation can be used to obtain the interplanar distance of a system: $n\lambda =2dsin\theta$, where n is considered to be 1, λ is 1.54178 Å. By using the interplanar distance, the lattice constant (*a*) for a cubic system is calculated as follows: $a=\sqrt{{d}^{2}({h}^{2}+{k}^{2}+{l}^{2})}$.

Raman spectra were measured by use of a Horiba Jobin Yvon-Labram Raman Microscope Spectrometer selecting the 488 nm excitation wavelength. Microbeam X-ray fluorescence (micro-XRF) spectra were measure by use of a custom-made instrument^80^. Attenuated total reflection-Fourier transform infrared (ATR-FTIR) analysis were carried out on a Spectrum Two, PerkinElmer spectrometer with a 4 cm^−1^ nominal resolution. The Transmission Electron micrographs were obtained using a high-resolution Titan THEMIS transmission electron Microscope operated at 200 kV.

### Luminescence measurements

The emission spectra were recorded using a wavelength tunable NT340 Series EKSPLA OPO (Optical Parametric Oscillator) operated at 10 Hz, with a pulse duration of 4 ns and laser spectral width of 5 cm^−1^. An intensified CCD (iCCD) camera (Andor Technology, iStar iCCD DH720) coupled to a spectrograph (Shamrock 303i, Andor) was used as detection system. An additional InGaAs photodiode array (Andor Technology, iDus DU490A) for monitoring the emission spectra in the 800–1700 nm range was used. The energy of the laser pulse was modified using neutral density filters and measured with a Coherent Energy Max Laser Energy Sensor (J-10MB-HE Energy Max Sensor). The energy of the laser pulse at 1533 nm varied from 0.5 up to 3.2 mJ. The X-ray induced luminescence spectra were measured by use of AvaSpec-HS1024x58/122TEC and AvaSpec-NIR256-1.7TEC fiber optic spectrometers. X-ray excitation source consists of an X-ray tube (Oxford Instruments, Apogee 5011, Mo target, max. high voltage −50 kV, max current −1 mA (<2 Gy/s). The digital photos were obtain by use of a Canon EOS 60D was used. For the near-infrared emission decay measurements, we used a NIR PMT module (H10330B-75, Hamamatsu) as detector coupled to a spectrograph/monochromator (Acton SP2758, Princeton Instruments) and a PCIe TCSPC card TimeHarp 260 NANO (PicoQuant) for acquisition. All lifetime estimations were calculated by integrating the area of the normalized at maximum emission decays.

## Electronic supplementary material

Supplementary Information

## Data Availability

The datasets generated during and/or analyzed during the current study are available from the corresponding author (CT) on reasonable request.
