# Supplementary material for: Near infrared emission properties of Er doped cubic sesquioxides in the second/third biological windows
Source: Sci Rep. 2018 Dec 21;8:18033. doi: 10.1038/s41598-018-36639-y (PMC6303399; doi:10.1038/s41598-018-36639-y)
Supplement: Supplementary file 1 — Supplementary Information [file 41598_2018_36639_MOESM1_ESM.pdf]

Supplemental Information

Near infrared emission properties of Er doped cubic sesquioxides in the second/third biological windows

Daniel Avram[a, b], Ion Tiseanu[a], Bogdan S. Vasile[c], Mihaela Florea[d] and Carmen Tiseanu[a, *]

[a]National Institute for Laser, Plasma and Radiation Physics, P.O. Box MG-36, RO 76900, Bucharest-Magurele, Romania

[b]University of Bucharest, Faculty of Physics, 405 Atomistilor Street, 077125 Magurele-Ilfov, Romania

[c]University POLITEHNICA from Bucharest, National Research Center for Food Safety, 313 Splaiul Independentei Street, RO 060042, Bucharest, Romania

[d]National Institute of Materials Physics, 405A Atomistilor Street, 077125 Magurele-Ilfov, Romania

[*]*Author to whom correspondence should be addressed. Electronic mail:* carmen.tiseanu@inflpr.ro

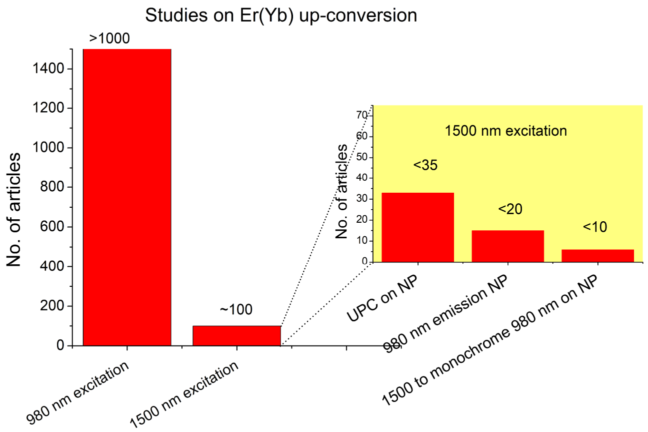

Figure S1. Comparison between the number of studies published in the last decade on up- conversion emission of Er(Yb) doped systems using 980 nm and ~1500 nm excitation wavelengths.

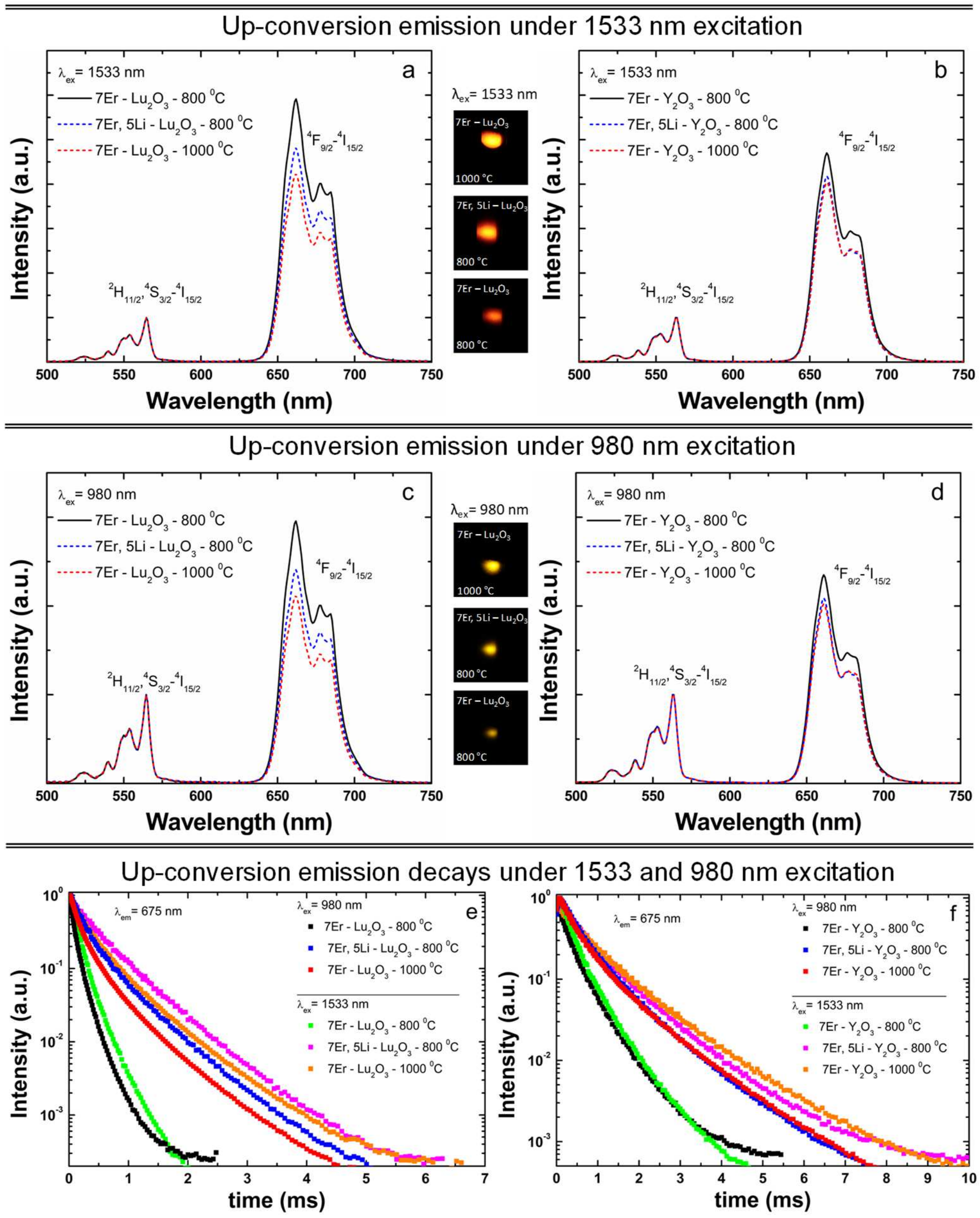

Figure S2. (a-d) Comparison of the up-conversion emission shape (spectra normalized at green emission) of 7Er – Lu_2O_3 (a, c) and 7Er – Y_2O_3 (b , d) series (with 5Li addition and increasing calcination temperature at 1000 °C) following 1533 (a, b) and 980 nm (c, d) excitation; (Middle) Digital images of 7Er – Lu_2O_3 – 800 °C; 7Er, 5Li – Lu_2O_3 – 800 °C and 7Er – Lu_2O_3 – 1000 °C nanopowders (sample holder area of 14 mm X 7 mm) taken in the *dark room light conditions* by use of Canon EOS 60D under exposure times of 1s with 400 ISO for under same optical setup and excitation energy, 1.8 ÷ 1.85 mJ. (e, f) Comparison of the up-conversion emission decays monitored at 675 nm of 7Er – Lu_2O_3 (e) and 7Er – Y_2O_3 (f) series following 1533 and 980 nm excitation.

Up-conversion emission under 1533 nm excitation yields an increase of RGR by 50-75 % compared to that under 980 nm excitation. This is likely due to the enhanced contribution of the $^4I_{13/2}$ energy level to the population of the $^4F_{9/2}$ red emitting level[1]. Usually, when increasing the nanoparticle size, the density of surface defects and thus their contribution on the nonradiative relaxation processes decreases, leading to a change of the emission shape, the enhancement of the luminescence intensity and the lengthening of the emission decays[2-5]. Similar to Y_2O_3 case (**Figure S2b, d**), the RGR measured under 1533 nm excitation are slightly lower for 7Er, 5Li – Lu_2O_3 (8.8) and for 7Er-Lu_2O_3 - 1000 °C (7.6) compared to the 7Er – Lu_2O_3 (10.5) (**Figure S2a, c**) due to diminished effect of the nonradiative relaxations from $^2H_{11/2}$, $^4S_{3/2}$ to $^4F_{9/2}$ energy level. Similar trend that is, decrease of RGR with particle size is observed for both Y_2O_3 and Lu_2O_3 samples under excitation at 980 and 1533 nm (**Figure S2b, c and d**)

Finally, the similar evolutions of the emission decays corresponding to red emitting level at 675 nm measures at both 980 and 1533 nm excitation (**Figure S2e and f**), suggests that the energy transfer mechanisms responsible for populating the red emitting energy level is mainly rooted from the long lived $^4I_{13/2}$ energy level.[5]

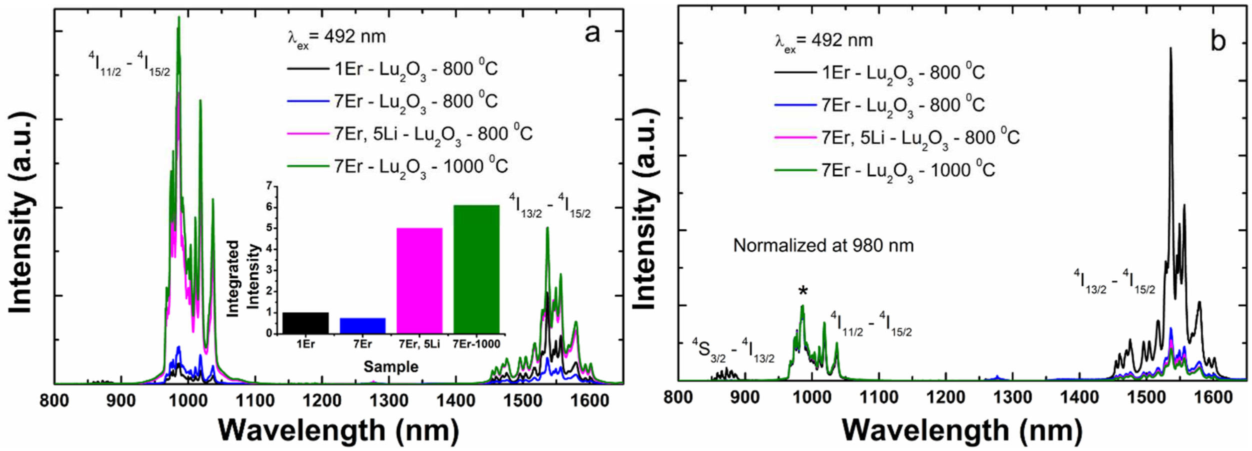

Figure S3. Effect of Er concentration and Li addition on the intensity (a) and shape (b) of down-conversion emission spectra of Er- Lu_2O_3 and Er- Lu_2O_3. Samples calcined at 1000 °C are included as reference samples (see text).

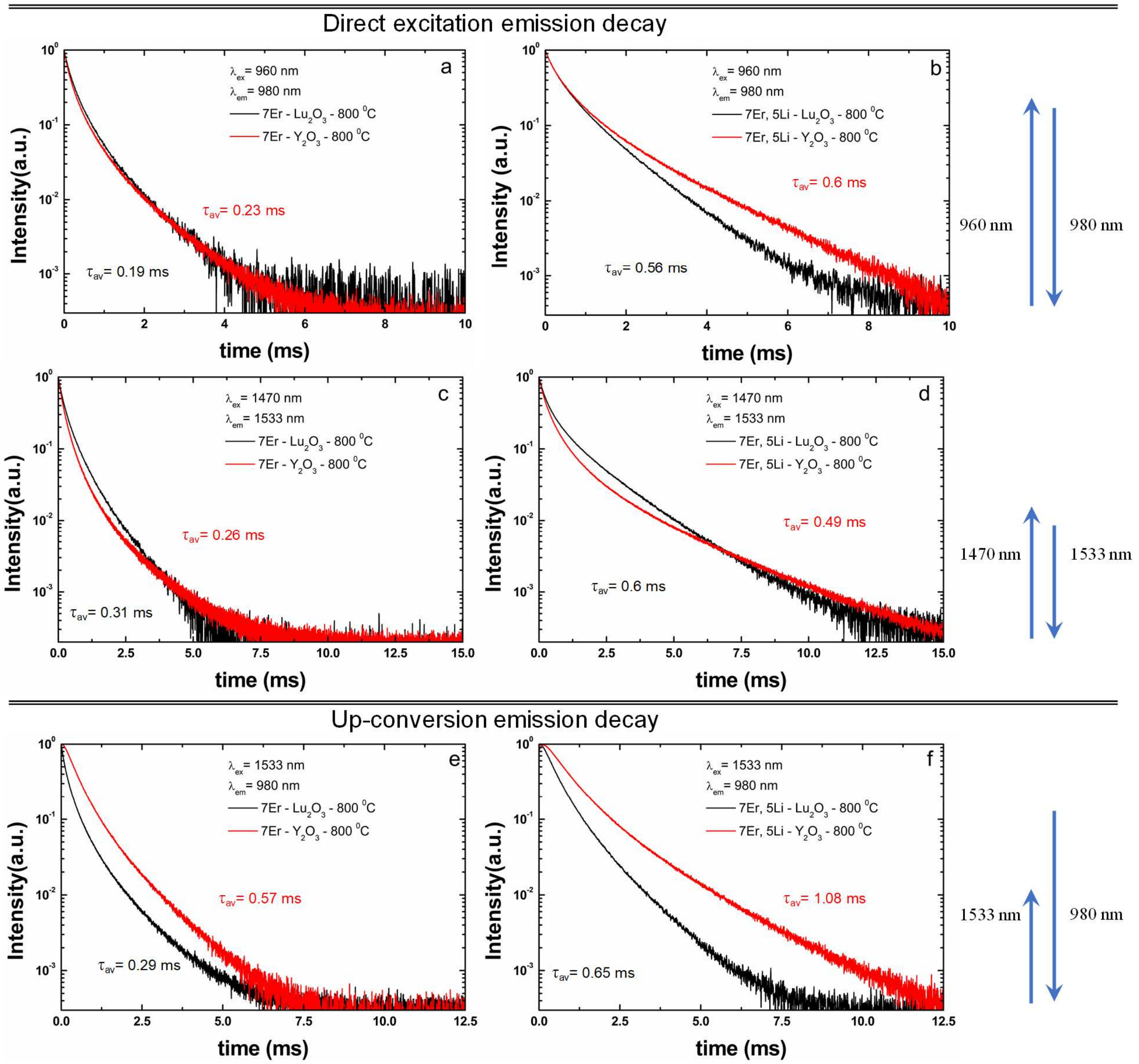

Figure S4. Comparison between direct excitation emission decays of 7Er - Lu_2O_3 - 800 °C and 7Er - Y_2O_3 - 800 °C (a, c) and 7Er, 5Li - Lu_2O_3 - 800 °C and 7Er, 5Li - Y_2O_3 - 800 °C (b, d) monitored at 980 nm (a, b) and 1533 nm (c, d) under 960 nm (a, b) and 1470 nm (c, d) excitation, respectively. Comparison between UPC emission decays of 7Er - Lu_2O_3 - 800 °C and 7Er - Y_2O_3 - 800 °C (e) and 7Er, 5Li - Lu_2O_3 - 800 °C and 7Er, 5Li - Y_2O_3 - 800 °C (f) monitored at 980 nm under 1533 nm excitation. *The samples (powders) were placed in the same geometrical configuration on a solid sample holder (sample holder area of 14 mm X 7 mm from Horiba Scientific, J1933) in reflection mode and excited with identical excitation energy (3.15 mJ for 960 nm excitation and 1.85 mJ for 1470 nm excitation).

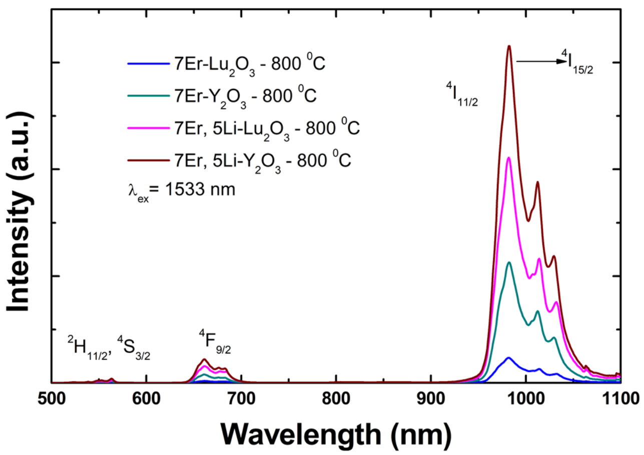

Figure S5. Comparison between up-conversion emission intensity of 7Er – (Lu_2O_3/Y_2O_3) - 800 °C and 7Er, 5Li – (Lu_2O_3/Y_2O_3) - 800 °C.

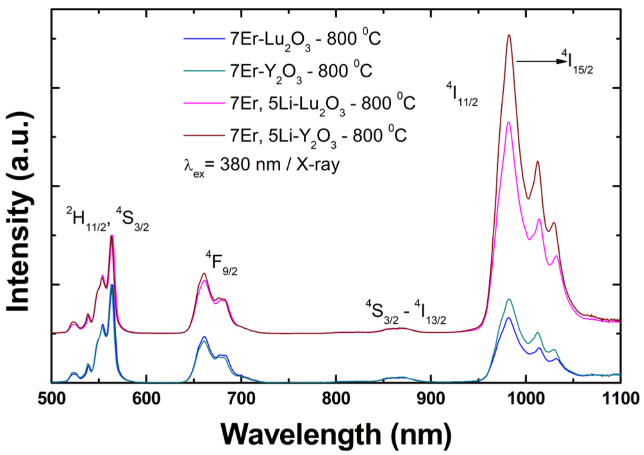

Figure S6. Comparison of X-ray induced emission (normalized at green emission intensity) of 7Er – (Lu_2O_3/Y_2O_3) - 800 °C and 7Er, 5Li – (Lu_2O_3/Y_2O_3) - 800 °C. *Following down-conversion excitation under 380 nm we observe the same emission spectra shape as for X-ray induced emission.

References:

1 Auzel, F. Upconversion and anti-stokes processes with f and d ions in solids. *Chemical Reviews* **104**, 139-173, (2004).

2 Zhao, J. *et al.* Upconversion luminescence with tunable lifetime in NaYF4:Yb,Er nanocrystals: role of nanocrystal size. *Nanoscale* **5**, 944-952, (2013).

3 Zheng, K., Song, W., Lv, C., Liu, Z. & Qin, W. Controllable synthesis and size-dependent upconversion luminescence properties of Lu2O3:Yb3+/Er3+ nanospheres. *Crystengcomm* **16**, 4329-4337, (2014).

4 Wang, F., Wang, J. & Liu, X. Direct Evidence of a Surface Quenching Effect on Size-Dependent Luminescence of Upconversion Nanoparticles. *Angewandte Chemie* **122**, 7618-7622, (2010).

5 Lu, H., Gillin, W. & Hernandez, I. Concentration dependence of the up- and down-conversion emission colours of Er3+-doped Y2O3: a time-resolved spectroscopy analysis. *Physical Chemistry Chemical Physics* **16**, 20957-20963, (2014).
